# Serum Per- and Polyfluoroalkyl Substances Are Associated with Increased Hearing Impairment: A Re-Analysis of the National Health and Nutrition Examination Survey Data

**DOI:** 10.3390/ijerph17165836

**Published:** 2020-08-12

**Authors:** Ming-Chieh Li

**Affiliations:** Department of Public Health, China Medical University College of Public Health, Taichung 40402, Taiwan; mingchiehli@mail.cmu.edu.tw

**Keywords:** per- and polyfluoroalkyl substances, PFAS, hearing impairment, hearing loss, NHANES

## Abstract

Although studies have shown that per- and polyfluoroalkyl substances (PFAS) are potential environmental ototoxicants, epidemiologic study has been limited. I conducted a cross-sectional study to re-examine the associations between PFAS and hearing impairment. Data were obtained from the National Health and Nutrition Examination Survey (NHANES) 1999–2000, 2003-06, 2009-12, and 2015-16. Perfluorooctanoic acid (PFOA), perfluorooctane sulfonic acid (PFOS), perfluorohexane sulfonic acid (PFHxS), and perfluorononanoic acid (PFNA) were measured in serum samples. Participants were divided into quartiles for each PFAS. Air conduction pure-tone audiometry was administered. Hearing impairment (1: yes, 0: no) was defined as a hearing threshold of more than 25 dB at 500, 1000, 2000, 4000, and 8000 Hz in the worse ear. I assessed the relation of serum PFAS with hearing impairment by the generalized linear mixed model with a logit link and binary distribution. Tests for linear trend across quartiles of serum PFAS were conducted using the median serum PFAS in each quartile as a continuous variable. After adjusting for age, sex, body mass index, education, ethnicity group, and family income, I found positive correlations between PFOA and hearing impairment at 2000 Hz (*p*-trend < 0.01) and 3000 Hz (*p*-trend = 0.02); between PFOS and hearing impairment at 500 Hz (*p*-trend < 0.01), 2000 Hz (*p*-trend < 0.0001) and 3000 Hz (*p*-trend = 0.02); between PFNA and hearing impairment at 2000 Hz (*p*-trend = 0.05), 3000 Hz (*p*-trend < 0.01), 4000 Hz (*p*-trend = 0.02), and 8000 Hz (*p*-trend < 0.01); between PFHxS and hearing impairment at 500 Hz (*p*-trend = 0.04), 1000 Hz (*p*-trend = 0.03), and 2000 Hz (*p*-trend < 0.01). However, some of the findings were not significant when only comparing the highest with the lowest quartile of PFASs. In conclusion, several background serum PFASs are positively correlated with hearing impairment in the United States adult population.

## 1. Introduction

According to the World Health Organization, an estimated 360 million people have some degree of hearing loss around the world [1], and exposure to loud noise is considered the major cause of impaired hearing function. A study has reported that about 24 percent of adolescents had audiometric notches, a sign of noise-induced hearing loss [2]. Several mechanisms have been proposed, and the major cause of noise-induced hearing loss is cochlear hair cell damage or synaptopathy. In addition, damage to cochlear neurons also contributes to noise-induced hearing loss [3]. In addition to noise-induced hearing loss, chemical exposure also plays an important role in inducing auditory impairment. Ototoxicity is defined as a substance that has the property of being toxic to the ear, affecting the inner ear or auditory nerve [4]. Although the literature has suggested that at least 750 different groups of chemicals are potential ototoxicants [5], few have been tested, and most of them were conducted in the occupational population with noise exposure [6]. How the ototoxicants affected the general population was less investigated. There is emerging evidence that environmental pollutants, such as persistent organic pollutants (POPs), are potential ototoxicants. POPs are a group of halogenated organic chemicals that do not break down easily and are therefore accumulated in the environment [7]. Only a few studies have indicated that POPs might adversely affect the hearing function [5]. Some studies further pointed out that in utero exposure to POPs might also be associated with hearing impairment [8,9]. This evidence suggested that the public should pay more attention to the potential ototoxicants. 

Per- and polyfluoroalkyl substances (PFAS) are listed as POPs, which are a group of manufactured chemicals that have been widely used in commercial products [10]. One study has shown that PFAS might be associated with hearing disorders [11]. The study has reported that the serum concentrations of perfluorooctanoic acid (PFOA) was higher in people who have trouble hearing (odds ratio (OR) = 1.40, 95% confidence interval (CI) = 1.01–1.93). Although the finding was of interest, the hearing disorder was assessed using a questionnaire. The study did not examine the auditory function among study participants. Recently, a study examined the associations between PFAS exposure and hearing impairment using the data from the National Health and Nutrition Examination Survey (NHANES) [12]. They concluded that there was no strong evidence to support the ototoxicity of PFAS exposure. However, the study somehow did not include the data from NHANES 1999–2000, which also contained per- and polyfluoroalkyl substances (PFAS) and audiometry data. In addition, although they found that no associations were observed between PFAS exposure and hearing impairment when comparing the participants with serum PFASs ≥ 90th versus <90th percentile, this kind of analysis might lack statistical power due to the relatively small sample size of the high exposure group. In addition, using participants with serum PFASs less than the 90th percentile as a reference group might underestimate the risk, because a lot of the participants in the reference group have middle and middle-high PFAS exposure. Findings from the analyses would likely weaken the exposure-outcome associations toward the null hypothesis. Therefore, I conducted a study to re-analyze the NHANES data and examine the associations between PFAS exposure and hearing impairment.

## 2. Materials and Methods

### 2.1. Participants

Ethical approval was not needed for this study because the data used in this study is publicly accessible (https://www.cdc.gov/nchs/nhanes/index.htm). I used data obtained from the NHANES, a nationally representative survey conducted by the US Centers for Disease Control and Prevention and the National Center for Health Statistics. NHANES includes measurements of nutritional status, chemicals and metabolites of environmental pollutants in the blood and urine, and health outcomes of the US general population, as described elsewhere [13]. The subsets of NHANES 1999–2000, 2003-06, 2009-12, and 2015-16 were used for assessing the association of serum PFAS with hearing impairment. Two subsets of NHANES were excluded due to lacking individual serum PFAS (NHANES 2001–2002) or audiometry data (NHANES 2007–2008). 

### 2.2. Exposure Assessment—Serum PFAS

In this study, four PFASs, including PFOA, perfluorooctane sulfonic acid (PFOS), perfluorohexane sulfonic acid (PFHxS), and perfluorononanoic acid (PFNA), were measured in serum samples. I only included these four PFASs, because they were detectable in more than 98% of participants. Automated solid-phase extraction coupled to isotope-dilution high–performance liquid chromatography–tandem mass spectrometry was used to measure serum PFASs [10,14]. 

### 2.3. Audiometric Measures

Standardized air conduction pure-tone audiometry was administered in a sound-isolated room by technicians trained by a certified audiologist. Air-conduction thresholds between 500 to 8000 Hz were tested for each ear using standard audiometric headphones. To ensure quality and reliability, the 1000 Hz frequency was tested twice in each ear [15]. Participants with cochlear implants, unable to tolerate headphones, or with abnormal otoscopy were excluded. Hearing impairment (1: yes, 0: no) was defined as a hearing threshold of more than 25 dB at 500, 1000, 2000, 4000, and 8000 Hz in the worse ear.

### 2.4. Statistical Analysis

Because serum PFASs and hearing threshold data were not a normal distribution, I categorized the data for all analyses. Participants were divided into quartiles for each PFAS. I assessed the relations of serum PFASs with hearing impairments using the logistic regression model, with PROC GLIMMIX procedure with a binomial distribution and logit link function. Tests for a linear trend across quartiles of serum PFASs were conducted using the median serum PFAS in each quartile as a continuous variable. Potential confounders were selected based on prior knowledge and their correlations with exposures and outcomes, including age, sex, body mass index, family poverty–income ratio, ethnicity, and education level. Participants with missing data were excluded. Sample weights were adjusted when performing statistical analyses [10]. I did not include the history of occupational or non-occupational noise exposure as potential confounders. Firstly, there was a considerable amount of data missing for these variables. Secondly and most importantly, there was no association between PFAS and occupational or non-occupational noise exposure as indicated by Ding and Park [12]. Since the definition of a confounder is “a third factor related to both the treatment and outcome might explain their association, with no true causal effect” [16], occupational or non-occupational noise exposure are not potential confounders in this study. A *p*-value less than 0.05 was considered statistically significant. All analyses were performed in SAS 9.4 software (SAS Institute, Cary, NC, USA). 

## 3. Results

Table 1 shows the demographic descriptions of the 2525 participants. Among each of our age categories, which were 20–29, 30–39, 40–59, and 60 years and older, the percentages were 20.0%, 19.6%, 33.9%, and 26.5%, respectively; more women (53.4%) than men; approximately 70% of participants had a BMI of 25 or higher; the majority of participants (55.0%) had an education level higher than college degree; most participants in our analysis were of non-Hispanic white ethnicity (40.2%), and; about 20% participants had a family income–poverty ratio less than 1, which means that the family income is less than the poverty threshold. 

The median concentrations and range of concentrations within each quartile of PFASs is shown in Table 2. The highest concentration of PFAS detected in this study was PFOS (median = 8.00 ng/mL), which ranged from 0.14 to 392 ng/mL; PFOA was the second most detected PFAS (median = 2.25 ng/mL), which ranged from 0.07 to 51.1 ng/mL.

Table 3 displays the associations between each serum PFAS and hearing impairment at different frequencies. After adjusting for age, sex, body mass index, education, ethnicity group, family income, and sample weights, positive associations were found between PFOA and hearing impairment at 2000 Hz and 3000 Hz. The odds ratios (ORs) for the highest compared with the lowest quartile of PFOA were 1.76 (95% confidence interval (CI) = 1.20–2.60, *p*-trend < 0.01) and 1.64 (95% CI = 1.16–2.34, *p*-trend = 0.02), respectively. Positive associations between PFOS and hearing impairment were found at 500, 2000, and 3000 Hz. The ORs for the highest compared with the lowest quartile of PFOS were 1.41 (95% CI = 0.93–2.17, *p*-trend < 0.01), 1.60 (95% CI = 1.09–2.37, *p*-trend < 0.0001), and 1.20 (95% CI = 0.85–1.71, *p*-trend = 0.02), respectively. Positive associations between PFNA and hearing impairment were found at 2000, 3000, 4000, and 8000 Hz. The ORs for the highest compared with the lowest quartile of PFNA were 1.68 (95% CI = 1.14–2.46, *p*-trend = 0.05), 1.52 (95% CI = 1.08–2.13, *p*-trend < 0.01), 1.53 (95% CI = 1.10–2.14, *p*-trend = 0.02), and 1.59 (95% CI = 1.16–2.17, *p*-trend < 0.01), respectively. Finally, I found positive associations between PFHxS and hearing impairment at 500, 1000, and 2000 Hz. The ORs for the highest compared with the lowest quartile of PFHxS were 1.26 (95% CI = 0.85–1.87, *p*-trend = 0.04), 1.44 (95% CI = 0.97–2.15, *p*-trend = 0.03), and 1.73 (95% CI = 1.19–2.52, *p*-trend < 0.01), respectively.

Sensitivity analyses were performed to examine if the new included dataset NHANES 1999–2000 affected the findings. I found similar results after excluding the NHANES 1999–2000 (data not shown in table). For example, a positive association was found between PFOA and hearing impairment at 2000 Hz. The odds ratio for the highest compared with the lowest quartile of PFOA was 1.76 (95% confidence interval (CI) = 1.20–2.60). After excluding the NHANES subset 1999–2000, the odds ratio was 1.71 (95% CI = 1.13–2.59). I also conducted a sensitivity analysis to adjust all the significant results for occupational noise exposure and found that there were only slight differences in the estimates (Supplemental Table S1).

## 4. Discussions

The results from a previous study [12] concluded that there is no strong association between PFAS exposure and hearing impairment. However, in the present study, I found that four PFASs, including PFOA, PFOS, PFNA, and PFHxS, were all associated with an increased risk of hearing impairments, although some of them were not significant when comparing the highest with the lowest quartile of PFASs. It seems that PFAS correlated with hearing impairment at almost all the frequencies except for 6000 Hz, which showed only borderline statistical significance with PFNA. Among them, the most consistent findings were the positive associations between the four PFASs (PFOA, PFOS, PFNA, and PFHxS) and hearing impairment at frequency 2000 Hz; followed by 3000 Hz, which was associated with three PFASs (PFOA, PFOS, and PFNA). The reason for the discrepancy might be due to the analysis strategies. The present study divided the participants into quartiles based on their serum concentrations of PFASs. Compared with the previous study conducted by Ding and Park [12], where they divided their study participants into two groups (serum PFASs ≥ 90th versus < 90th percentile), we can perform comparisons between participants with relatively high and low concentrations of PFASs. Moreover, we might have more statistical power to detect the difference because of the reasonable sample size in the highest exposure group.

In addition to Ding and Park’s study [12], only one epidemiologic study has been published that assessed the association between PFASs and possible hearing impairment [11]. In that study, the authors examined the effects of several environmental pollutants on the self-reported hearing condition and found that participants who had trouble hearing might have higher serum concentrations of PFOA (3.0 ± 2.0 ng/mL), compared with those who had good hearing (2.5 ± 2.2 ng/mL). However, no association was found between PFOS, PFNA, PFHxS, and the other PFASs with trouble hearing [11]. The current study confirmed the findings that PFOA is associated with hearing problems, although inconsistent findings were also found between PFOS, PFNA, and PFHxS and hearing problems. The reason for the inconsistencies might be due to the methodology of assessing hearing problems. Shuie’s study assessed the overall hearing problems by a self-reported questionnaire. The author did not examine the frequency-specific hearing thresholds. In the current study, I found possible hearing impairments mostly at frequencies of 2000 and 3000 Hz, suggesting that some hearing frequencies might be more vulnerable to PFASs.

Although previous animal studies have reported that some environmental pollutants might be potential ototoxicants, such as dioxins, polychlorinated biphenyl, and phthalates [5], the studies on PFAS were limited. One study in rats examined the developmental neurotoxicity of potassium PFOS and found no effect on auditory startle responsiveness [17]. Another study in rats assessed the effect of perfluorobutyrate (PFBA) but also found no effects on hearing ability [18]. However, unlike studies of the other environmental contaminants, the aforementioned two studies did not assess hearing audiometry; the hearing problems might not be detected if there was only mild hearing impairment at a specific frequency. Although lacking biological evidence, an experimental study showed that PFOA affected intracellular Calcium ions (Ca2^+^) homeostasis in hippocampal neurons (Liu et al., 2011). Loss of the sensorineural cells, including outer hair cells, has been known as a key factor contributing to hearing impairment [19]. Increasing evidence has shown that intracellular Ca2^+^ homeostasis may be a contributory factor in making outer hair cells [20]. 

Fábelová and colleagues have also postulated three possible mechanisms for the associations between environmental chemicals and hearing impairments. Firstly, the thyroid effect. Several environmental pollutants might interfere with thyroid function, which in turn can induce neurodevelopmental toxicity. PFASs have been identified as possible thyroid toxicants [21]. Second, oxidative stress induced hearing impairment. Oxidative stress has been confirmed as one of the major causes of hearing impairment [22]. Many environmental pollutants, including PFASs [23,24], were found to be associated with increased oxidative stress. Finally, the interaction with the aryl hydrocarbon receptor (AhR): The AhR is a ligand-activated transcription factor that controls xenobiotic detoxification through the induction of cytochrome P450 1A1 [25]. Activation of AhR leads to the upregulation of uridine diphosphate glucuronosyltransferases, resulting in hypothyroxinemia during a critical period of cochlear development, which in turn affects the development of hair cells and hearing ability. PFASs are known ligands of the AhR [26,27] and thus might induce hearing impairment through this pathway.

The strengths of this study include a fair sample size to assess the possible association between PFASs and auditory function. This study offers epidemiologic evidence that background PFAS exposure may be correlated with hearing impairment among the US adult population. The most important limitation of the study is its cross-sectional design, so the causality cannot be established. In addition, I only have a one-time assessment of the serum PFASs and pure-tone audiometry, which may introduce a misclassification of the exposures and outcomes. Finally, animal studies that can provide additional support to my findings are lacking.

In conclusion, the current study found positive associations of PFOA, PFOS, PFNA, and PFHxS with hearing impairment, and the frequencies 2000 Hz and 3000 Hz were most affected. Due to the nature of the cross-sectional design, the observed findings need to be verified in more prospective cohort studies. Further animal and experimental studies are warranted to elucidate the underlying mechanism. 

## Figures and Tables

**Table 1:** The demographic characteristics of study participants.

Variables	*N* = 2525
**Age (years)**	
20–29	506 (20.0%)
30–39	495 (19.6%)
40–59	855 (33.9%)
≥60	669 (26.5%)
**Gender**	
Female	1349 (53.4%)
Male	1176 (46.6%)
**Body Mass Index**	
<18.5	29 (1.2%)
18.5–24.99	718 (28.4%)
25–29.99	825 (32.7%)
≥30	953 (37.7%)
**Education level**	
Less Than 9th Grade	237 (9.4%)
9–11th Grade (Includes 12th grade with no diploma)	342 (13.5%)
High School Grad/GED or Equivalent	550 (21.8%)
Some College or AA degree	786 (31.1%)
College Graduate or above	610 (24.2%)
Family poverty-income ratio *	
<1	512 (20.3%)
1–4.99	1560 (61.8%)
≥5	453 (17.9%)
**Race/Ethnicity**	
Mexican American	412 (16.3%)
Other Hispanic	259 (10.3%)
Non-Hispanic White	1016 (40.2%)
Non-Hispanic Black	517 (20.5%)
Other Race	321 (12.7%)

* Family income–poverty ratio less than 1 means that the family income is less than the poverty threshold; GED: General Educational Development; AA degree: associates degree.

**Table 2:** The median concentrations and range of concentrations within each quartile of PFASs.

	Range of Each Quartile				
	Q1	Q2	Q3	Q4	Median
PFOA	0.07–1.36	1.37–2.24	2.25–3.61	3.62–51.1	2.25
PFOS	0.14–4.02	4.03–7.98	8.0–17.0	17.1–392	8.00
PFNA	0.06–0.49	0.50–0.79	0.80–1.20	1.21–19.4	0.8
PFHS	0.07–0.70	0.71–1.29	1.30–2.28	2.29–36.5	1.3

Unit: ng/mL.

**Table 3:** The associations between four PFASs and hearing impairment (hearing threshold levels >25 dB) (*N* = 2525).

	Hearing Threshold						
PFAS	500	1K	2K	3K	4K	6K	8K
PFOA							
Q1	Referent	Referent	Referent	Referent	Referent	Referent	Referent
Q2	0.83 (0.54–1.27)	1.21 (0.79–1.85)	1.41 (0.95–2.10)	1.39 (0.98–1.98)	1.31 (0.95–1.83)	1.08 (0.82–1.43)	1.24 (0.93–1.66)
Q3	0.89 (0.58–1.35)	1.22 (0.80–1.85)	1.26 (0.85–1.87)	1.38 (0.98–1.96)	1.12 (0.81–1.56)	1.08 (0.81–1.44)	0.87 (0.65–1.18)
Q4	1.06 (0.70–1.60)	1.21 (0.80–1.85)	**1.76** (1.20–2.60)	**1.64** (1.16–2.34)	**1.41** (1.01–1.98)	1.16 (0.86–1.56)	0.87 (0.64–1.20)
P-trend	0.36	0.58	<0.01	0.02	0.11	0.38	0.11
PFOS							
Q1	Referent	Referent	Referent	Referent	Referent	Referent	Referent
Q2	0.83 (0.53–1.31)	0.77 (0.49–1.22)	0.70 (0.46–1.06)	0.76 (0.53–1.08)	0.69 (0.50–0.97)	0.99 (0.75–1.31)	1.03 (0.77–1.37)
Q3	0.91 (0.59–1.39)	1.07 (0.71–1.63)	1.12 (0.76–1.65)	1.00 (0.71–1.41)	0.89 (0.65–1.24)	1.14 (0.86–1.51)	0.98 (0.72–1.32)
Q4	1.41 (0.93–2.17)	1.16 (0.76–1.77)	**1.60** (1.09–2.37)	1.20 (0.85–1.71)	1.02 (0.73–1.44)	1.11 (0.82–1.50)	0.99 (0.72–1.35)
P-trend	<0.01	0.13	<0.0001	0.02	0.14	0.42	0.85
PFNA							
Q1	Referent	Referent	Referent	Referent	Referent	Referent	Referent
Q2	1.53 (0.99–2.34)	1.43 (0.93–2.19)	**1.61** (1.09–2.39)	1.12 (0.80–1.58)	1.28 (0.93–1.77)	1.09 (0.83–1.44)	**1.35** (1.01–1.82)
Q3	1.23 (0.81–1.90)	1.36 (0.90–2.07)	1.29 (0.87–1.90)	0.98 (0.70–1.37)	1.12 (0.81–1.55)	1.03 (0.78–1.37)	**1.49** (1.10–2.00)
Q4	1.37 (0.90–2.10)	1.39 (0.91–2.12)	**1.68** (1.14–2.46)	**1.52** (1.08–2.13)	**1.53** (1.10–2.14)	1.30 (0.97–1.75)	**1.59** (1.16–2.17)
P-trend	0.45	0.33	0.05	<0.01	0.02	0.09	<0.01
PFHxS							
Q1	Referent	Referent	Referent	Referent	Referent	Referent	Referent
Q2	0.79 (0.52–1.21)	1.08 (0.72–1.65)	1.13 (0.76–1.68)	1.30 (0.91–1.85)	0.89 (0.63–1.24)	0.95 (0.72–1.27)	1.06 (0.78–1.42)
Q3	0.89 (0.59–1.34)	0.96 (0.63–1.44)	1.21 (0.83–1.77)	1.29 (0.91–1.82)	0.87 (0.62–1.21)	1.01 (0.76–1.35)	1.11 (0.82–1.50)
Q4	1.26 (0.85–1.87)	1.44 (0.97–2.15)	**1.73** (1.19–2.52)	**1.44** (1.01–2.05)	0.93 (0.66–1.31)	0.94 (0.70–1.28)	0.84 (0.61–1.15)
P-trend	0.04	0.03	<0.01	0.10	0.96	0.74	0.14

Data were presented as odds ratios and 95% confidence interval. Models were all adjusted for age, sex, body mass index, education, ethnicity group, family income, and sample weights.

## Supplementary Materials

The following are available online at https://www.mdpi.com/1660-4601/17/16/5836/s1, Table S1: The associations between four PFASs and hearing impairment (hearing threshold levels >25 dB) (*N* = 2309).
