# Supplementary material for: Serum Per- and Polyfluoroalkyl Substances Are Associated with Increased Hearing Impairment: A Re-Analysis of the National Health and Nutrition Examination Survey Data
Source: Int J Environ Res Public Health. 2020 Aug 12;17(16):5836. doi: 10.3390/ijerph17165836 (PMC7460726; doi:10.3390/ijerph17165836)
Supplement: Supplementary file 1 [file ijerph-17-05836-s001.pdf]

Supplemental materials

Table 1. The associations between four PFASs and hearing impairment (hearing threshold levels >25dB) (*N* = 2309).

				Hearing Threshold			
**PFAS**	**500**	**1K**	**2K**	**3K**	**4K**	6K	8K
				PFOA			
Q1			Referent	Referent			
Q2			1.29 (0.86–1.93)	1.36 (0.95–1.94)			
Q3			1.25 (0.84–1.87)	1.42 (1.00–2.03)			
Q4			**1.68** (1.12–2.52)	**1.87** (1.29–2.69)			
P-trend			0.01	<0.01			
				PFOS			
Q1	Referent		Referent	Referent			
Q2	0.83 (0.52–1.31)		0.71 (0.46–1.08)	0.77 (0.54–1.10)			
Q3	0.90 (0.58–1.40)		1.05 (0.71–1.56	1.01 (0.71–1.43)			
Q4	1.36 (0.87–2.13)		**1.70** (1.14–2.56)	1.40 (0.97–2.03)			
P-trend	0.02		<.0001	<0.01			
				PFNA			
Q1			Referent	Referent	Referent		Referent
Q2			**1.82** (1.19–2.77)	1.24 (0.87–1.78)	1.31 (0.93–1.84)		**1.45** (1.06–1.97)
Q3			1.42 (0.93–2.15)	1.01 (0.71–1.45)	1.15 (0.82–1.62)		**1.66** (1.22–2.28)
Q4			**1.86** (1.23–2.80)	**1.67** (1.17–2.38)	**1.60** (1.13–2.26)		**1.77** (1.27–2.46)
P-trend			0.03	<0.01	0.02		<0.01
				PFHxS			
Q1	Referent	Referent	Referent				
Q2	0.76 (0.48–1.19)	1.02 (0.65–1.58)	1.06 (0.70–1.60)				
Q3	0.87 (0.57–1.34)	1.06 (0.69–1.62)	1.17 (0.79–1.73)				
Q4	1.32 (0.87–1.99)	**1.53** (1.01–2.32)	**1.65** (1.12–2.44)				
P-trend	0.02	0.01	<0.01				

Data were presented as odds ratios and 95% confidence interval. Models were all adjusted for age, sex, body mass index, education, ethnicity group, family income, sample weights, and occupational noise exposure.
